# Expression of B2 Receptor on Circulating CD34-Positive Cells and Outcomes of Myocardial Infarction

**DOI:** 10.1155/2019/7816438

**Published:** 2019-07-08

**Authors:** Cong Fu, Yuhan Cao, Yuyu Yao, Shengxing Tang, Qun Fan, Yang Ling

**Affiliations:** ^1^Department of Cardiology, Yi Ji Shan Hospital Affiliated to Wan Nan Medical College, China; ^2^Key Laboratory of Non-Coding RNA Transformation Research of Anhui Higher Education Institution (Wan Nan Medical College), China; ^3^Department of Nephrology, Yi Ji Shan Hospital Affiliated to Wan Nan Medical College, China; ^4^Department of Cardiology, Zhong Da Hospital Affiliated to Southeast University, China

## Abstract

**Background:**

Bradykinin B2 receptor (B2R) is a widely expressed cell surface receptor. The relationship between B2R expression on circulating CD34+ cells and prognosis of myocardial infarction remains unknown.

**Methods:**

We analyzed the expression of B2R on circulating CD34-positive cells and plasma VEGF concentration in 174 myocardial infarction patients. All involved patients were divided into two groups: high B2R group and low B2R group according to the median B2R expression percentage. 48 months of follow-up was performed. The endpoints were heart failure and revascularization.

**Results:**

The plasma level of VEGF in the low B2R group is 67 ± 12 pg/mL, whereas the high B2R group has significantly elevated VEGF levels of 145 ± 27 pg/mL (*P* < 0.001). The concentration of VEGF has correlated with expression of B2R (*r* = 0.574, *P* < 0.001). During the 48 months of follow-up, low expression of B2 receptor on circulating CD34-positive cells indicates the high incidence of heart failure (hazard ratio: 2.247; 95% confidence interval: 1.110-4.547; *P* = 0.024) and revascularization (hazard ratio: 2.335; 95% confidence interval: 1.075-5.074; *P* = 0.032). Kaplan-Meier survival analysis showed that the cumulative hazard of heart failure (*P* = 0.014) and revascularization (*P* = 0.032) has significant differences between low B2R and high B2R.

**Conclusion:**

Low expression of B2R on circulating progenitor cells indicated the poor outcomes of myocardial infarction.

## 1. Introduction

Cardiovascular disease is the major cause of death worldwide that frequently leads to irreversible heart failure [1]. Accompanied by the development of drug and invasive treatment, the mortality of ST-elevated myocardial infarction (STEMI) is lower than in the past. Despite the pathological process and inflammatory factors, the risk factors that influence the outcomes of STEMI remained not totally clear [2, 3].

Stem and progenitor cells derived from bone marrow get into circulating blood immediately when ischemic injury occur [4–7]. Stem and progenitor cells participated in repairing post-STEMI. Previous studies revealed that endothelial progenitor cells (EPCs) are capable of angiogenesis and differentiating into endothelial cells which are candidates for vascular regeneration [8, 9]. It can be concluded that the mobilization of stem cells and progenitor cells benefits the outcomes of ischemic damage. CD34 is a widely known marker of progenitor cells. CD34-positive cells can be found in peripheral blood [10]. However, it is unknown if CD34 have a relationship with the outcomes of myocardial ischemia damage.

B2 receptor (B2R) is the receptor of bradykinin which is important for the effect of bradykinin and is expressed on several cells including some progenitor cells, for example, EPCs. B2R has a key role in the angiogenesis [11]. We suggest a hypothesis that expression of B2R on circulating CD34-positive cells has the relationship to the prognosis of myocardial infarction.

## 2. Methods

### 2.1. Study Population

Between October 2013 and December 2014, 174 STEMI patients who consecutively underwent coronary angiography in Yi Ji Shan Hospital affiliated to Wan Nan Medical College were involved in the study. The definition of myocardial infarction was described previously [12]. Patients who have cancer, stroke, old myocardial infarction, severe liver dysfunction, chronic renal disease, heart failure (Killip III-IV), autoimmune diseases, and infection diseases, received stent implantation before, are receiving hormone and/or immunosuppressant therapy and hemodialysis, and died before discharge from the hospital were excluded. Also, lack of clinical document was excluded. All patients were asked to confirm their agreement to accept the 48 months of follow-up by providing written informed consent. This trial design was approved by the Ethics Committee in Yi Ji Shan Hospital (Approval number: 2013YJYSYLL108.1) and performed according to the Declaration of Helsinki.

### 2.2. Arterial Blood Sample Collected and Analyzed

Before revascularization, 20 mL of arterial blood sample was collected in EDTA-coated tubes. Mononuclear cells were isolated on a Lymphocyte Separation Solution (HaoYang, China) by centrifugation at 500 × g for 20 min according to standard protocols. Plasma was collected in EP tubes and stored at -40°C. The plasma VEGF level was determined by a Human VEGF ELISA Kit (BOSTER, Wuhan, China).

### 2.3. Flow Cytometry

**Figure 1:**
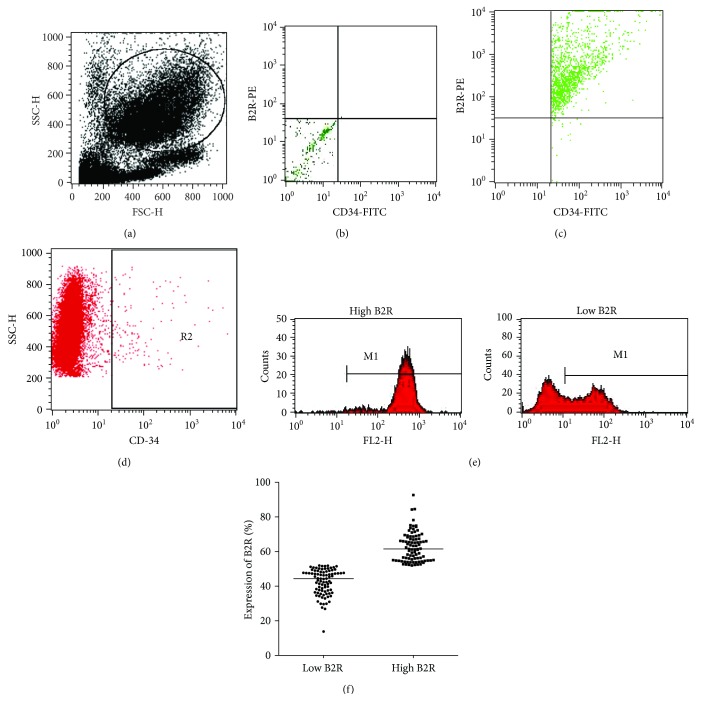
Representative figure of flow cytometry analysis: (a) the gating for mononuclear cells; (b) the negative control; (c) the CD34- and B2R-positive control; (d) R2 represents CD34-positive cells in all gating cells. (e) Representative FACS figure of B2R expression in two groups. M1 represents B2R-positive cells. (f) Scatter diagram shows the expression percentage of B2R between the low B2R and high B2R groups.

For flow cytometry analysis, mononuclear cells were suspended in 200 *μ*L of phosphate-buffered saline (PBS) containing 1% bovine serum albumin (BSA). Afterward, they were fixed in 1% paraformaldehyde and then permeabilized using 0.1% Triton X-100 containing 0.5% BSA. The cells were then incubated with specific rabbit anti-human B2R antibodies (Abcam, UK) on ice for 1 hour, followed by incubation with a PE-conjugated donkey anti-rabbit secondary antibody (Santa Cruz, USA) and a fluorescein isothiocyanate- (FITC-) conjugated anti-human CD34 antibody (BD Biosciences, USA) for 1 hour. Stained cells were washed 3 times with PBS and then analyzed using a FACScan flow cytometer (Becton Dickinson, USA) to detect B2R expression on CD34-positive cells (Figure 1). Firstly, mononuclear cells were gated. Secondly, the CD34-positive cells were gated; then, the B2R-positive cells were further analyzed. The proportion of B2R-positive cells among CD34-positive cells was calculated. The positive control and negative control were presented.

### 2.4. Angiography

Cardiac catheterization was performed according to the guidelines for coronary angiography of the American College of Cardiology and the American Heart Association [13]. The Gensini score was recorded according to a previous study [14].

### 2.5. Therapy Procedures

All the patients that participated in the study underwent coronary angiography, and 112 patients underwent percutaneous coronary intervention (PCI). Usage of platelet inhibitors or anticoagulants and other symptomatic treatments was left to the discretion of the treating physician according to the guideline and clinical condition. Clopidogrel and aspirin were administered at 600 mg and 300 mg after arrival in the hospital; and the patients were immediately transferred to the catheter room where CAG and/or PCI were performed. Clopidogrel and aspirin were administered at 75 mg and 100 mg per day after PCI, respectively. Beta receptor blocker, statin, and LMWH were administered according to the patients' status and guideline. Angiotensin-converting enzyme inhibitors/angiotensin receptor blocker (ACEI/ARB) was administered in patients who have a serum creatinine level less than 256 *μ*mol/L.

### 2.6. Endpoints

Major adverse cardiovascular events (MACEs) were recorded. Coronary revascularization was defined as angioplasty, stenting, or coronary artery bypass grafting during the follow-up. Heart failure was defined as BNP measured in Yi Ji Shan Hospital at least one value above the 5∗99th percentile upper reference limit and having clinical symptoms in hospitalization during the follow-up. MACEs were verified by hospital medical records and telephone. No missing data was generated during the follow-up.

### 2.7. Statistical Analysis

The data were analyzed using the statistical software package of SPSS (SPSS Inc., Chicago, IL, USA, Version 17.0). Numerical variables were expressed as the mean ± standard deviation and categorical variables as percentages. Continuous variables between groups were compared by unpaired Student's *t*-test. Categorical variables were compared by the Chi-squared test. Kaplan-Meier survival analysis was performed. The hazard ratio (HR) and 95% confidence interval (CI) were calculated by the Cox proportional hazards model. Two-tailed *P* values < 0.05 were considered significant.

## 3. Results

### 3.1. The Number of Circulating CD34-Positive Cells and Expression of B2R

Flow cytometry showed that among the 174 patients, the proportion of circulating CD34-positive cells ranged from 0.89% to 1.36% (0.98 ± 0.12%). The expression of B2R on circulating CD34-positive cells ranged from 13.8% to 92.6% (median: 53.2%). The expression of B2R between two groups is shown in Figures 1(e) and 1(f).

### 3.2. Baseline Characteristics

According to the median level (53.2%) of B2R expression on CD34-positive cells, the patients were divided into two groups: low B2R (≤53.2%, *n* = 87) and high B2R group (>53.2%, *n* = 87). Detailed characteristics of the patients are listed in Table 1. The age, biochemical data, degree of coronary artery stenosis, and drug therapy were matched. WBC counts were associated with high B2R expression on CD34-positive cells (*P* < 0.001). The influence of clinical properties was further analyzed by multivariate analyses.

### 3.3. The Plasma Level of VEGF

**Figure 2:**
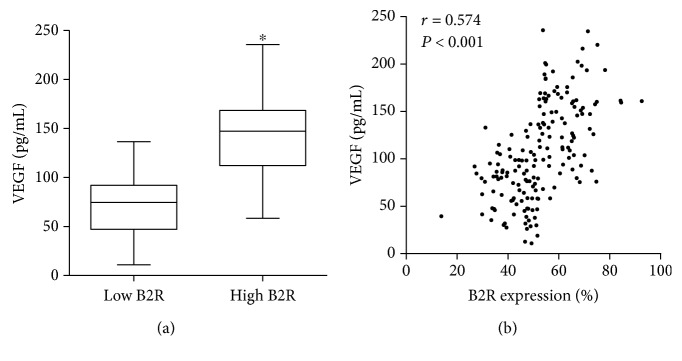
Plasma VEGF concentration of two groups and relationship with B2R expression. (a) Box plot shows the VEGF concentration of two groups. (b) Pearson correlation between the low and high B2R groups (^∗^*P* < 0.001).

As shown in Figure 2, the plasma level of VEGF in the low B2R group is 67 ± 12 pg/mL, whereas the high B2R group has significantly elevated VEGF levels (145 ± 27 pg/mL, *P* < 0.001) (Figure 2(a)). Pearson correlation analysis showed that the serum concentration of VEGF is correlated with B2R expression (*r* = 0.574, *P* < 0.001) (Figure 2(b)).

### 3.4. Incidence of MACEs

In univariate analyses, 25 patients in the low B2R group and 9 patients in the high B2R group have heart failure, respectively (HR: 2.501; 95% CI: 1.166-5.364; *P* = 0.018). Revascularization occurred in 26 patients in the low B2R group and 12 patients in the high B2R group, respectively (HR: 2.066; 95% CI: 1.042-4.096; *P* = 0.038).

In multivariate analyses, after adjusting for the age, sex, WBC, LM stenosis, PCI, plasma VEGF concentration, and drug treatment, the adjusted HR for heart failure is 2.247 (95% CI: 1.110-4.547, *P* = 0.024) and for revascularization is 2.335 (95% CI: 1.075-5.074, *P* = 0.032) (Table 2). The age, sex, WBC, LM stenosis, PCI, and plasma VEGF concentration are eliminated by the Cox proportional hazards model.

**Figure 3:**
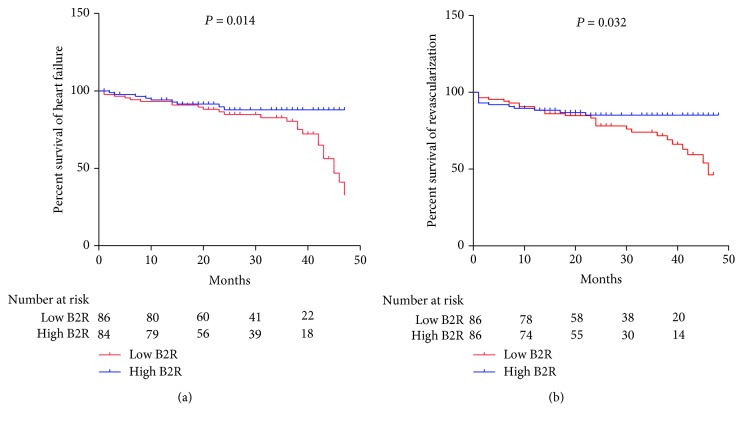
Kaplan-Meier survival analysis of MACEs: (a) percentage survival of heart failure between two groups; (b) percentage survival of revascularization between two groups.

Kaplan-Meier survival analysis showed that the cumulative hazard of heart failure (*P* = 0.014) and revascularization (*P* = 0.032) has significant differences between the two groups (Figure 3).

## 4. Discussion

Our study indicated that low expression of B2R on circulating CD34-positive cells has a strong relation to the poor outcomes of STEMI. It is the first statement that the expression of B2R on progenitor cells has influenced the outcomes of ischemic damage.

CD34 is expressed on hematopoietic stem/progenitor cells as an adhesion molecule and disappeared gradually during the maturation of these cells. Progenitor cells own the ability to differentiate into several cells. Many experimental and clinical types of research showed that progenitor cells are involved in vascular repairing [15]. In particular, the transplantation of progenitor cells has clinical benefits in the treatment of vascular injury [16]. Moreover, Werner et al. found that the level of circulating CD34^+^KDR^+^ EPCs predicts the occurrence of cardiovascular events and death from cardiovascular causes. However, the predictability of the circulating CD34-positive cell number for the occurrence of cardiovascular events has yet to be reported in other cardiovascular diseases. According to a previous study [10] and this research, the proportion of CD34-positive cells is below 2%. In addition, if circulating CD34-positive cells have the ability to predict the outcomes of STEMI remains unknown. Thus, it is needed to find another way to identify the relation between circulating progenitor cells and outcomes in STEMI.

B2R is a member of the G protein-coupled receptor superfamily and a critical cell surface receptor molecule that is activated by BK and expressed on numerous cells, including EPCs that regulate cell proliferation and injury repair [11]. Spinetti et al. [17] had found that a B2R-dependent mechanism is involved in the invasive capacity of EPCs activated by tissue kallikrein which indicated that the B2R signal pathway is essential for the protective effect of EPCs. Previous researches have also shown that the B2R-mediated signaling pathway has suppressed the cell dysfunction through recruitment of circulating CD34-positive cells [11, 18]. Further, low B2R expression is associated with inhibition of cell proliferation [19]. In an animal model, age-related low B2R protein levels may leave the heart vulnerable to ischemic damage [20]. Recently, our previous study further demonstrated that B2R regulate the oxidative stress-induced premature senescence of EPCs [21]. In addition, B2R is strongly associated with P53 expression, as B2R knockout diabetic mice are resistant to oxidative stress-induced mitochondrial injury and show high expression of the tumor suppressor gene P53 [22]. In the ischemia disease model, activation of the B2R-associated signal pathway inhibits the development of myocardial infarction [23]. Besides, myocardial ischemia triggers B2 receptor upregulation in both the infarcted and noninfarcted areas of the heart [24]. Our data found that low B2R expression of circulating CD34-positive cells indicates the poor outcomes of STEMI. The possible reason is that high B2R expression triggers the protective effect of the B2R-associated signal pathway that further mobilizes the circulating EPCs.

VEGF is a classic factor that mediates angiogenesis [25]. Previous research indicated that plasma VEGF concentration is related to coronary collateral function in patients with CHD [26]. However, VEGF worked in many ways. The activation of B2R can further trigger the receptor of VEGF that promotes the angiogenesis. On the other side, VEGF may lead to plaque instability as a proinflammation factor. Our study showed that the expression of B2R is related to plasma VEGF concentration. However, the Cox proportional hazards model eliminates plasma VEGF concentration. VEGF is not an effective single biomarker that indicates the prognosis of coronary artery disease. The poor outcomes in low B2R patients may have a relation to the poor angiogenesis after myocardial infarction.

Further, EPCs are a major source of CD34-positive cells. A previous study suggested that EPC transplantation has the potential to cure myocardial infarction via the activated B2R signal pathway [27]. The results from our cohort study suggested that low B2R led to poor outcomes. We can further explain that low B2R expression failed to activate the B2R signal pathway when EPCs which mobilized to peripheral blood cannot be developed to exert its protective effects such as promoting cell proliferation and angiogenesis.

This research has several limitations. Firstly, the sample size is small, so the reliability of the results needs to be validated by a large sample trial. Secondly, the CD34 is not the unique marker of progenitor cells such as EPCs. Thirdly, if the increased incidence rate of MACEs is related to the failed activation of B2R that EPCs cannot develop its protective effect needs to be further studied by basic fundamental research.

## 5. Conclusion

Low expression of B2R on circulating progenitor cells indicated the poor outcomes of STEMI.

## Figures and Tables

**Table 1:** Baseline characteristics of myocardial infarction patients.

	Patients (*n* = 174)		*P*
	Low B2R (*n* = 87)	High B2R (*n* = 87)	
Sex (M/F)	68/19	70/17	0.500
Age (y)	69 ± 10	69 ± 11	0.718
WBC	7.2 ± 2.0	9.5 ± 3.9	<0.001
RBC	4.4 ± 0.5	4.5 ± 0.6	0.284
HB	133 ± 17	134 ± 17	0.972
Gensini score	81 ± 16	86 ± 23	0.684
B2R expression (%)	31.1 ± 4.6	76.5 ± 9.3	0.617
VEGF (pg/mL)	67 ± 12	145 ± 27	<0.001
cTnI	6.2 ± 2.7	14.0 ± 7.6	0.109
TC	4.3 ± 1.0	4.3 ± 1.2	0.058
TG	1.4 ± 0.7	1.3 ± 0.6	0.594
LDL	2.6 ± 0.8	2.7 ± 0.9	0.134
HDL	1.1 ± 0.2	1.1 ± 0.4	0.082
eGFR	78 ± 30	76 ± 34	0.775
Smoking, *n* (%)	26 (29.9)	17 (19.5)	0.080
HP, *n* (%)	71 (81.6)	65 (74.7)	0.180
DM, *n* (%)	26 (29.9)	26 (29.9)	NS
LM, *n* (%)	3 (3.4)	9 (10.3)	0.132
LAD, *n* (%)	53 (60.9)	58 (66.7)	0.528
LCX, *n* (%)	39 (44.8)	39 (44.8)	NS
RCA, *n* (%)	38 (43.7)	42 (48.3)	0.648
PCI, *n* (%)	53 (60.9)	59 (67.8)	0.429
Aspirin, *n* (%)	82 (94.3)	83 (95.4)	NS
Betaloc, *n* (%)	64 (73.6)	65 (74.7)	NS
ACEI/ARB, *n* (%)	49 (56.3)	48 (55.2)	NS
Statin, *n* (%)	76 (87.4)	79 (90.8)	0.628
LMWH, *n* (%)	45 (51.7)	52 (59.8)	0.360
Clopidogrel, *n* (%)	55 (63.2)	65 (74.7)	0.140

WBC: white blood cell (∗10^9^/L); HB: hemoglobin (g/L); Neutrophil: ∗10^9^/L; cTnI: cardiac troponin I (ng/mL); RBC: red blood cell (∗10^12^/L); TC: total cholesterol (mmol/L); TG: triglyceride (mmol/L); LDL: low-density lipoprotein (mmol/L); HDL: high-density lipoprotein (mmol/L); eGFR: estimate glomerular filtration rate (mL/min/1.73m^2^); HP: hypertension; DM: diabetes mellitus; LM: Left Main Artery; LAD: left anterior descending branch; LCX: Left Circumflex Artery; RCA: right coronary artery; PCI: percutaneous coronary intervention; ACEI/ARB: angiotensin-converting enzyme inhibitors/angiotensin receptor blocker; LMWH: low molecular weight heparins; NS: *P* = 1.000.

**Table 2:** Multivariate analysis of the association between expression of B2R and outcomes.

Outcomes	Low B2R	High B2R	HR (95% CI)	*P*	Adjusted HR (95% CI)	*P*
Heart failure no./total no.	25/87	9/87	2.501 (1.166-5.364)	0.018	2.247 (1.110-4.547)	0.024
%	28.7	10.3				
Revascularization no./total no.	26/87	12/87	2.066 (1.042-4.096)	0.038	2.335 (1.075-5.074)	0.032
%	29.9	13.8				

HR: hazard ratio; CI: confidence interval.

## Data Availability

The datasets used and/or analyzed in the current study are available from the corresponding author on reasonable request.
